# Supplementary figures and images for: Characterizing transcriptomic responses to sediment stress across location and morphology in reef-building corals
Source: PeerJ. 2024 Jan 30;12:e16654. doi: 10.7717/peerj.16654 (PMC10836209; doi:10.7717/peerj.16654)

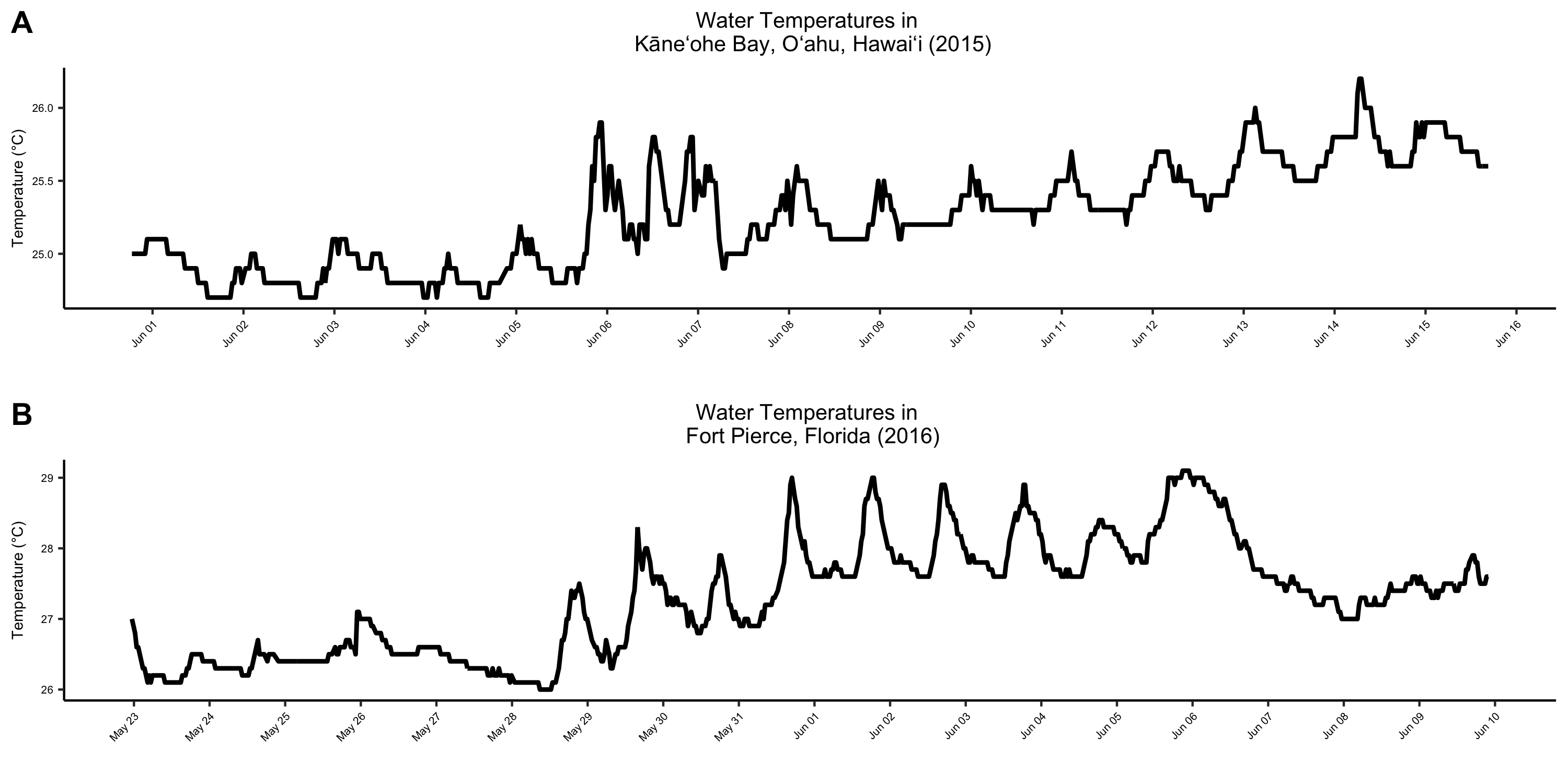

Supplement: Supplemental Information 1 — (A) Sea surface temperature in Kāne‘ohe Bay, O‘ahu, Hawai‘i from June 1st to June 15th, 2015. Data obtained from Station 51207, Kāne‘ohe Bay, HI (NOAA National Data Buoy Center). (B) Sea surface temperature in Fort Pierce, Florida from May 23rd to June 9th, 2016. Data obtained from Station 41114, Fort Pierce, FL (NOAA National Data Buoy Center). [file peerj-12-16654-s001.png]

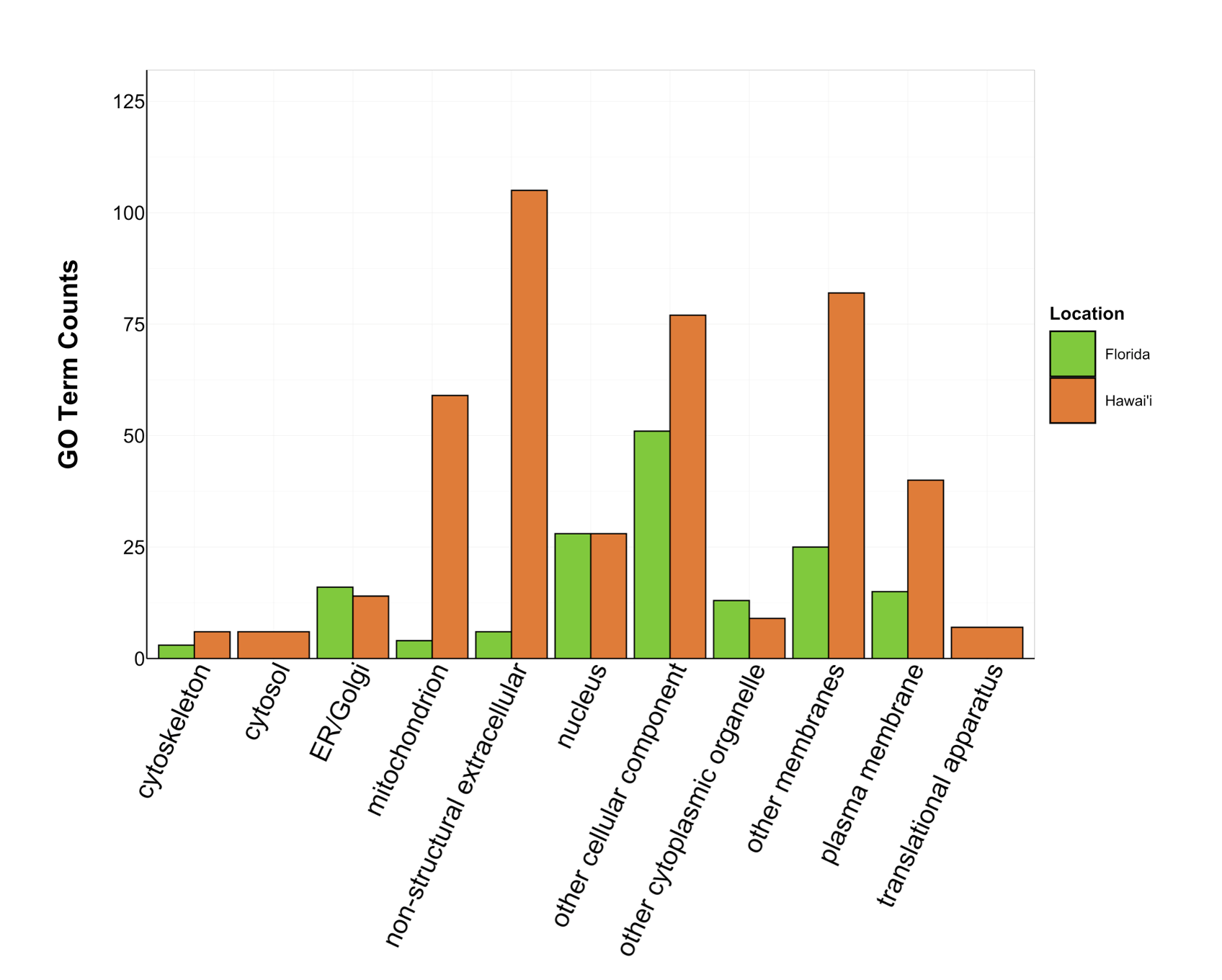

Supplement: Supplemental Information 2 — GO slim categories are on the x-axis, while the number of Cellular Component GO terms in each GO slim category is on the y-axis. The bars are colored by location: green bar = Florida, orange bar = Hawai’i. [file peerj-12-16654-s002.png]

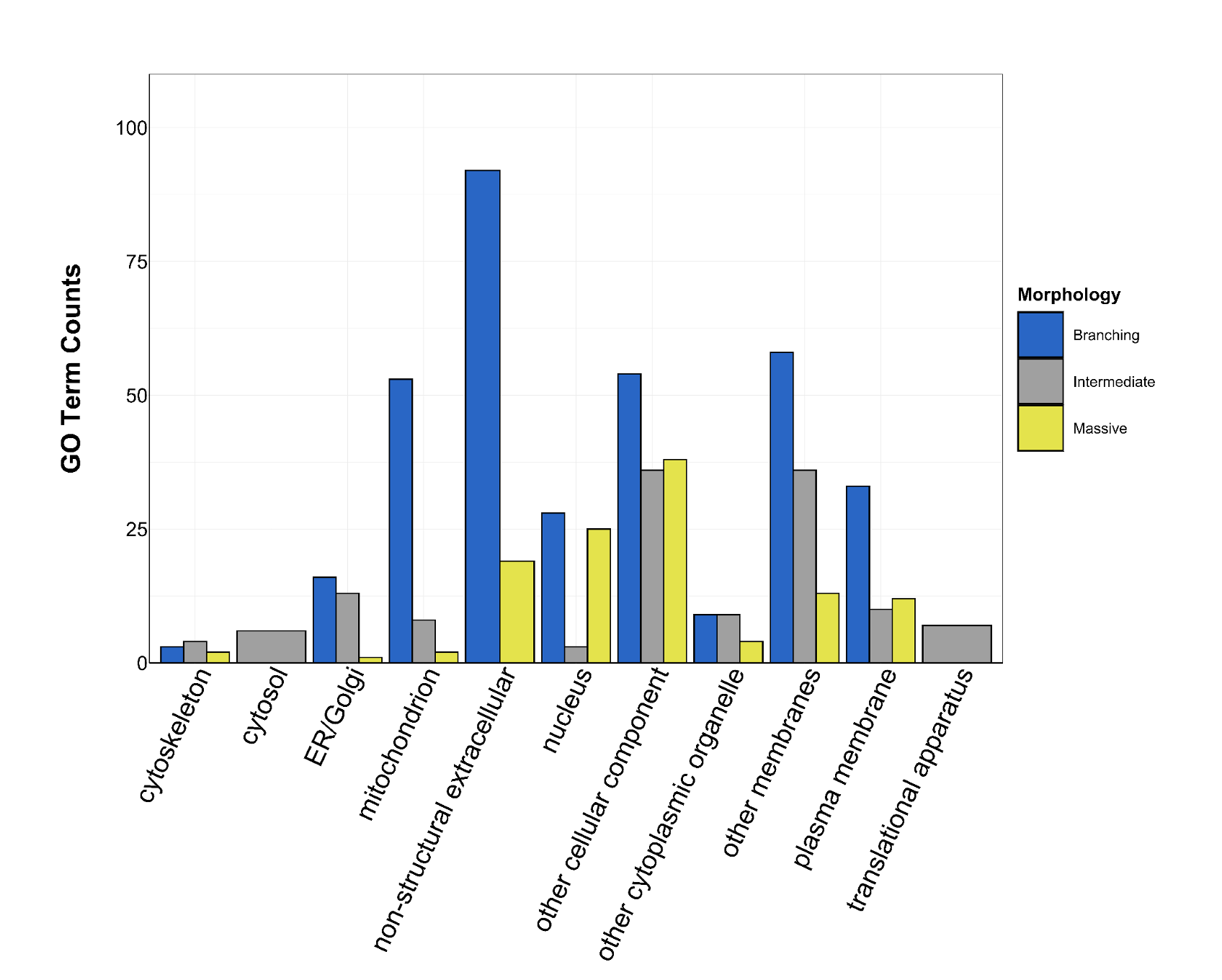

Supplement: Supplemental Information 3 — GO slim categories are on the x-axis, while the number of Cellular Component GO terms in each GO slim category is on the y-axis. The bars are colored by morphology: blue bar = branching, gray bar = intermediate, yellow bar = massive. [file peerj-12-16654-s003.png]

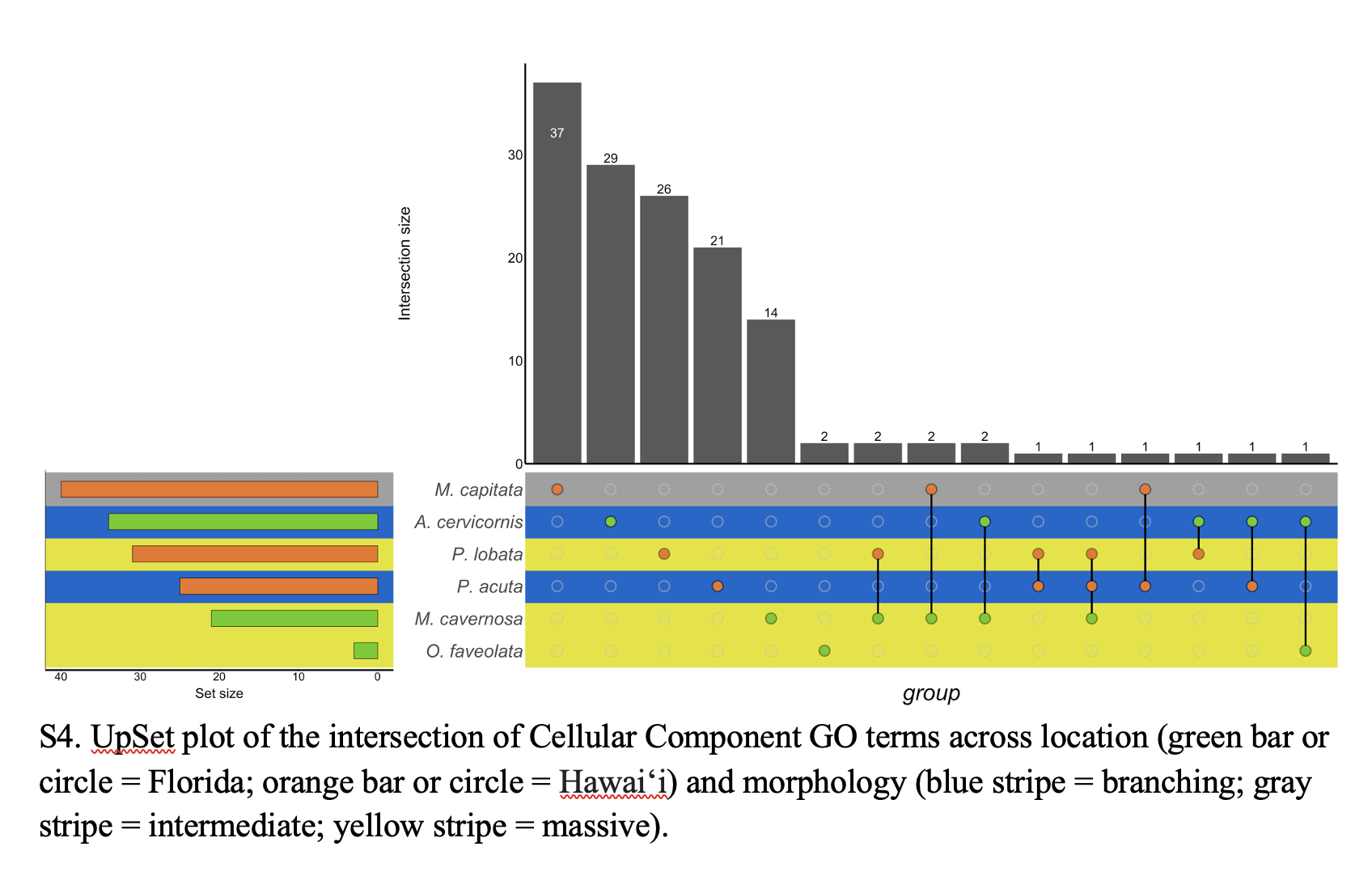

Supplement: Supplemental Information 4 — Green bar or circle corresponds to Florida; orange bar or circle corresponds to Hawai‘i. Blue strip corresponds to branching morphology; yellow stripe corresponds to massive morphology. Gray stripe corresponds to intermediate morphology. [file peerj-12-16654-s004.png]

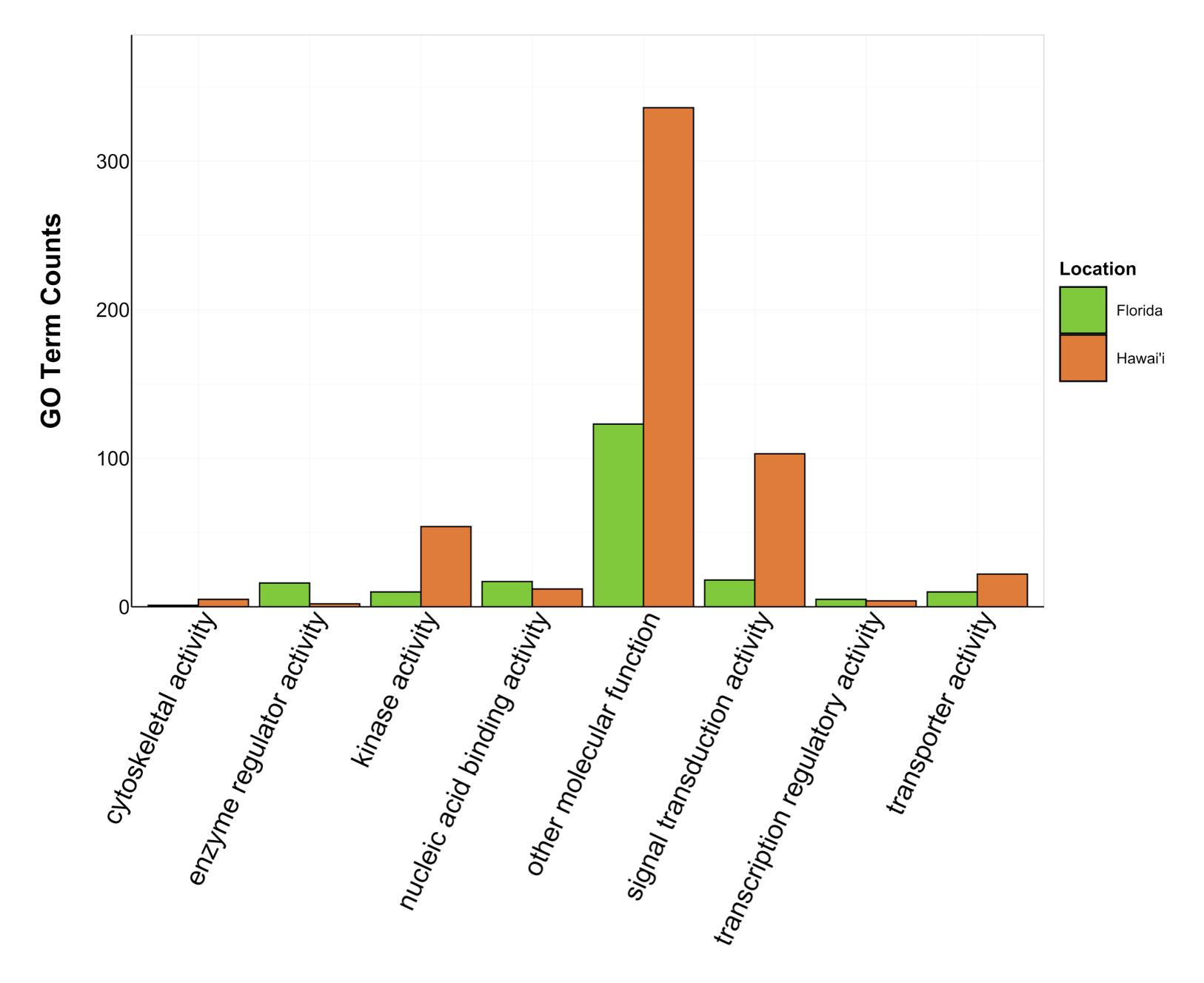

Supplement: Supplemental Information 5 — GO slim categories are on the x-axis, while the number of Molecular Function GO terms in each GO slim category is on the y-axis. The bars are colored by location: green bar = Florida, orange bar = Hawai’i. [file peerj-12-16654-s005.png]

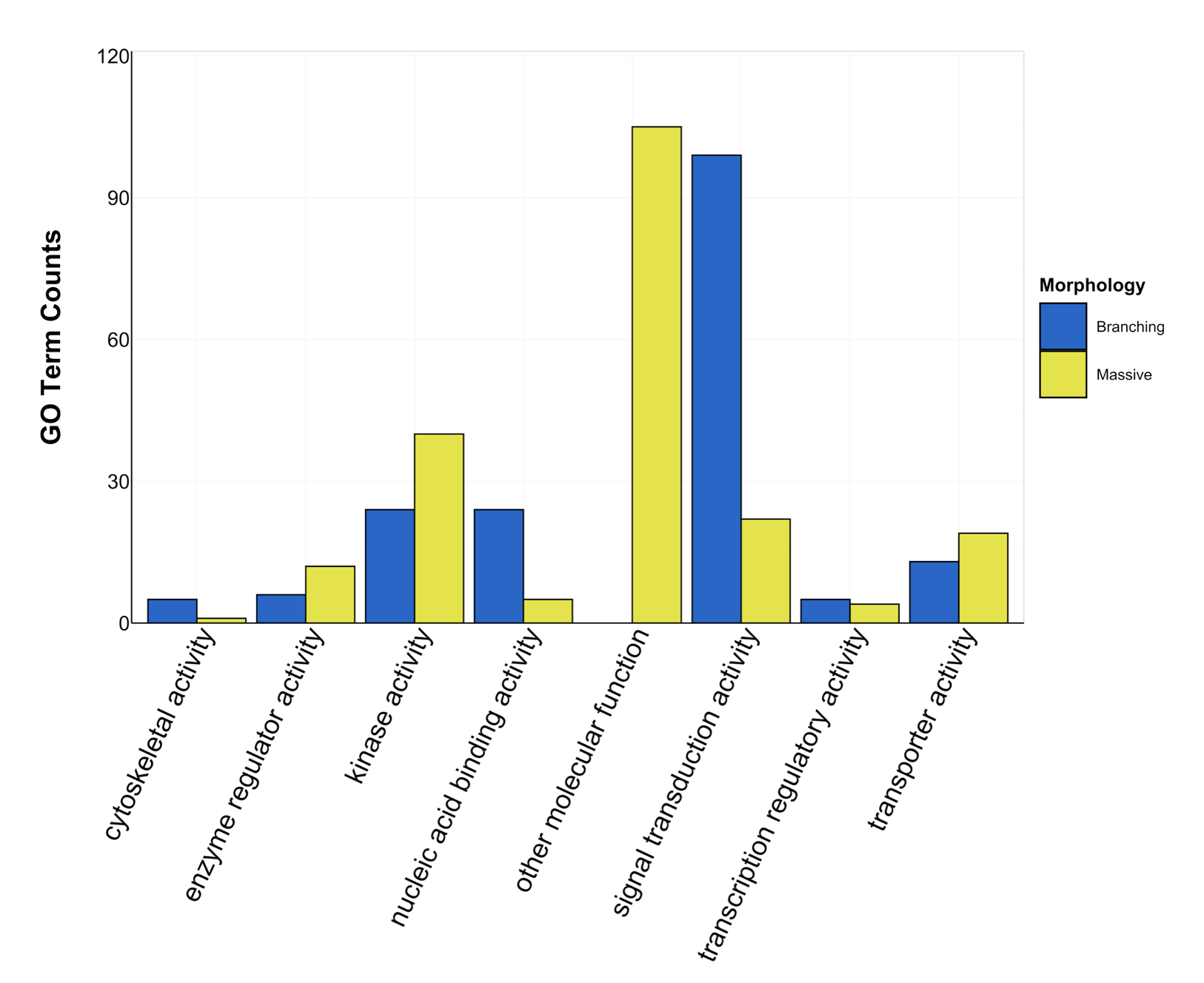

Supplement: Supplemental Information 6 — GO slim categories are on the x-axis, while the number of Molecular Function GO terms in each GO slim category is on the y-axis. The bars are colored by morphology: blue bar = branching, yellow bar = massive. Intermediate morphology was not assigned any Molecular Function GO terms. [file peerj-12-16654-s006.png]

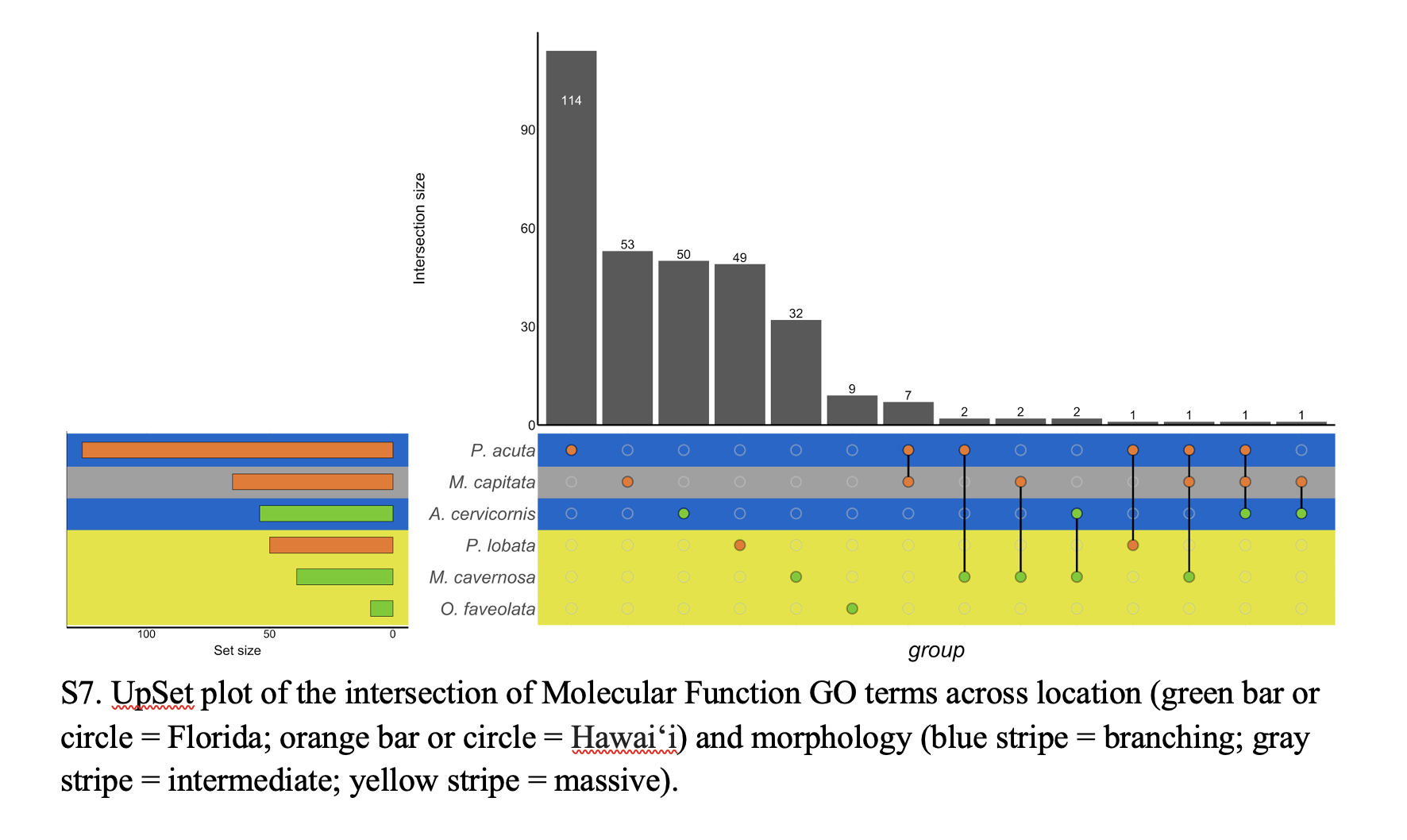

Supplement: Supplemental Information 7 — Green bar or circle corresponds to Florida; orange bar or circle corresponds to Hawai‘i. Blue strip corresponds to branching morphology; yellow stripe corresponds to massive morphology. Gray stripe corresponds to intermediate morphology. [file peerj-12-16654-s007.png]
